# Comparison of bilateral and unilateral upper limb training in people with stroke: A systematic review and meta-analysis

**DOI:** 10.1371/journal.pone.0216357

**Published:** 2019-05-23

**Authors:** Pei-ming Chen, Patrick W. H. Kwong, Claudia K. Y. Lai, Shamay S. M. Ng

**Affiliations:** 1 Department of Rehabilitation Sciences, The Hong Kong Polytechnic University, Hong Kong, China (SAR); 2 School of Nursing, The Hong Kong Polytechnic University, Hong Kong, China (SAR); Shanghai Jiao Tong University Affiliated Sixth People's Hospital, CHINA

## Abstract

**Background and objectives:**

Bilateral upper limb training (BULT) and unilateral upper limb training (UULT) are two effective strategies for the recovery of upper limb motor function after stroke. This meta-analysis aimed to compare the improvements in motor impairment and functional performances of people with stroke after BULT and UULT.

**Research design and methods:**

This systematic review and meta-analysis identified 21 randomized controlled trials (RCTs) met the eligibility criteria from CINAHL, Medline, Embase, Cochrane Library and PubMed. The outcome measures were the Fugl-Meyer Assessment of Upper Extremity (FMA-UE), Wolf Motor Function Test (WMFT), Action Research Arm Test (ARAT) and Box and Block Test (BBT), which are validated measures of upper limb function.

**Results:**

Twenty-one studies involving 842 subjects with stroke were included. Compared with UULT, BULT yielded a significantly greater mean difference (MD) in the FMA-UE (MD = 2.21, 95% Confidence Interval (CI), 0.12 to 4.30, p = 0.04; I^2^ = 86%, p<0.001). However, a comparison of BULT and UULT yielded insignificant mean difference (MD) in terms of the time required to complete the WMFT (MD = 0.44; 95%CI, -2.22 to 3.10, p = 0.75; I^2^ = 55%, p = 0.06) and standard mean difference (SMD) in terms of the functional ability scores on the WMFT, ARAT and BBT (SMD = 0.25; 95%CI, -0.02 to 0.52, p = 0.07; I^2^ = 54%, p = 0.02).

**Discussion and implications:**

Compared to UULT, BULT yielded superior improvements in the improving motor impairment of people with stroke, as measured by the FMA-UE. However, these strategies did not yield significant differences in terms of the functional performance of people with stroke, as measured by the WMFT, ARAT and BBT. More comparative studies of the effects of BULT and UULT are needed to increase the reliability of these conclusions.

## Introduction

Stroke often causes upper limb motor function deficits. Accordingly, people with stroke tend to be more reliant on others during their daily lives [1]. Severe motor impairments in the upper limbs were found to persist for 6 months after stroke in a third of people with stroke [2]. More than half of the activities of daily living (e.g. dressing, feeding and cooking) rely on upper limb functions [3]. Therefore, motor impairment in the upper limb presents a significant barrier to reintegration into society [4].

Unilateral upper limb training (UULT), a common rehabilitative strategy for people with stroke, includes repetitive task-related training [5–7]and constraint-induced movement training (CIMT) [8–13]. During CIMT, the subjects are required to wear a constraint mitten on the unaffected upper limb and to perform intensive training with affected side for at least 6 hours per day. Compared with CIMT, task-related training is a less intensive form of goal-directed training. This form uses various types of motor tasks to help subjects to derive optimal control strategies for solving problems related to motor abilities. Wolf et al. [8]compared the effects of a 14-session CIMT program with dose-matched conventional therapy (day treatment program, outpatient visits, physiotherapy and occupational therapy) on the motor outcomes of people with sub-acute stroke. In that study, CIMT induced a significantly greater increase in the Quality of Movement in Motor Activity Log (MAL) score (p<0.01) and a significant reduction in the time needed to complete the Wolf Motor Function Test (WMFT) (p<0.01), compared to the conventional treatment program. Additionally, task-related training was also found to be superior to conventional therapy for rehabilitating upper limb function. Narayan et al. [7]found that 20 sessions of task-related upper limb training, including reaching and lifting objects with different shapes using the affected upper limb, were superior to dose-matched neurodevelopmental-based therapy as measured by the Fugl-Meyer Assessment of Upper Extremity (FMA-UE), WMFT, MAL and Action Research Arm Test (ARAT).

Bilateral upper limb training (BULT) is another stroke motor rehabilitation strategy in which the subjects are required to perform motor tasks with both upper limbs. Here, the unimpaired limb is used to increase the functional recovery of the impaired limb by facilitating coupling effects between the two limbs [14]. BULT includes bilateral functional task training [15–19], bilateral robotic-assisted training [20–22] and bilateral arm training with rhythmic cueing [23–25]. Several studies have indicated the superiority of BULT over various conventional therapies (including neurodevelopmental therapy[18, 26], occupational therapy [19, 27, 28], physiotherapy [17, 21, 28] and unilateral robotic-assisted training [20]) for improving the FMA-UE, WMFT, ARAT and MAL and the ranges of motion (ROM) of the shoulder, elbow and wrist joints in people with stroke.

Several clinical trials have also demonstrated the ability of BULT to improve hemiplegic arm functions [29–33]. In two systematic reviews [29, 32], combinations of BULT with other therapies, such as electrical stimulation and auditory rhythmic cueing, effectively increased the functional WMFT scores of patients with acute to chronic stroke immediately after completion of the intervention. However, those reviews [29, 32] included single-group pre-post studies [34–39]. Moreover, the reviews [29, 32] did not directly compare the results of BULT and UULT in people with stroke and thus, were unable to demonstrate which approach more effectively improved the performance of the paretic upper limb, based on functional scales such as the FMA-UE, WMFT and ARAT.

By contrast, two recently published meta-analyses [33, 40] compare the abilities of BULT and UULT to improve the FMA-UE, WMFT, ARAT and MAL in people with stroke. Van Delden et al. [33] categorized studies according to the motor impairment level, as measured by the FMA-UE, Brunnstorm Stage, WMFT and ARAT. The results showed that UULT and BULT yielded similar improvement in the FMA-UE, WMFT, ARAT and MAL scores of people with stroke. Lee et al. [40] compared the effects of BULT with those of unilateral task-related training and CIMT during upper limb rehabilitation after stroke. Notably, CIMT was more effective than BULT in improving the WMFT and ARAT score. However, this finding should be interpreted cautiously because only three studies [16, 41, 42] were included in the meta-analysis.

Although CIMT can be used to train the paretic upper limb intensively, a direct comparison of the effects of CIMT and BULT on the rehabilitation of upper limb motor function in people with stroke may be inappropriate. First, CIMT requires the subjects to wear a constraint mitten on the unaffected upper limb and to perform intensive training for at least 6 hours per day [43]. Page et al. [44] found that about 68% of subjects with stroke were unable to complete the full schedule of CIMT because of the training requirement and restrictive device. According to Blanton et al. [45], only 20–25% of patients with chronic stroke benefited from CIMT because of the tight training schedule and potential risk induced by the restricted training plan. By contrast, BULT has a lower training intensity. Patients are expected to complete approximately 1 to 2 hours of training per session on 3 to 5 days per week [24, 46], in contrast to the CIMT schedule of 6 hours of supervised task practice on each of 14 consecutive days [10, 47, 48]. Second, most CIMT studies applied stringent inclusion criteria, including at least 10 degrees of wrist extension, thumb abduction and finger extension on the affected side [49]. Compared with CIMT, BULT only requires people with stroke to maintain volitional control of the non-paretic arm, to be capable of flexing the paretic arm and shoulder and to have maintained the residual grip function of the paretic hand [18, 25]. CIMT is only applied to stroke survivors with mild to moderate levels of upper limb dysfunction. Thus, the exclusion of CIMT from a comparison of the effects of BULT and UULT in people with stroke would improve the validity of the quantitative results.

Although several meta-analyses [33, 40] have compared the effects of BULT and UULT in people with stroke according to the FMA-UE, WMFT and ARAT, these meta-analyses treated CIMT as a subtype of UULT and included it in comparison with BULT. To the best of our knowledge, no studies have excluded CIMT when comparing the effects of BULT and UULT in people with stroke. This systematic review and meta-analytical review aimed to evaluate the available randomized controlled trials (RCTs) that compared the effects of BULT and UULT, but excluding CIMT on improvements in the FMA-UE, WMFT and ARAT score of people after stroke.

## Methodology

### Study selection criteria

An exhaustive search of the literature was conducted to identify publications related to the effectiveness of BULT. The CINAHL, Medline, Embase, Cochrane Library and PubMed databases were searched systematically through April 2018 using the keywords listed in Table 1.

**Table 1:** Keywords used in the search strategy.

	Key Words
AND	stroke OR CVA OR cerebrovascular disease OR cerebrovascular accident OR hemiparesis OR hemiplegia OR paresis
AND	bilateral OR unilateral OR BATRAC OR bilateral arm training with rhythmic auditory cueing OR bimanual OR bilateral robotic
AND	upper limb OR upper extremity OR arm OR forearm OR wrist OR finger OR hand
AND	randomized OR randomized controlled trial OR RCT

All identified full-text English language journal articles were screened independently by the two reviewers (PM and PK). The reference lists of the selected articles were then examined to identify additional potential articles. The inclusion criteria were applied to identify studies that (1) were randomized control trials; (2) reported quantitative behavior outcome measures; (3) had investigated the effects of interventions on upper limb function; (4) included an intervention group with bilateral movement training; (5) an intervention group with unilateral movement training or conventional occupational therapy or physiotherapy; and (6) included people with stroke. The following exclusion criteria were also applied: (1) the use of BULT in both the experimental and control groups; (2) failure to provide data on the outcome measures; (3) the studies with was a single session design; (4) systematic review or meta-analyses; and (5) inclusion of CIMT as the UULT.

### Risk of bias

The methodological quality of the studies was assessed using the Cochrane Collaboration tool for assessing the risk of bias [50]. Studies that provided information clearly (i.e., randomized, subject-blinded) were rated as low risk for the corresponding items. If the study provided the information against the assessed items (i.e. non-randomized, not blinded), the study was rated as high risk for those items. If no information suitable for our judgment process was provided, the study was rated as unclear.

### Data synthesis and analysis

Two reviewers (PM and PK) extracted the participants’ demographic information (age, gender, post-stroke duration post stroke, type of lesion and side of hemiparesis), details of the intervention (type, intensity and duration) and outcome measures to identify the study characteristics. The third reviewer (SMN) made judgments if discrepancies occurred between the two reviewers. The International Classification of Functioning, Disability and Health (ICF), which is regarded as the international standard for evaluations of health and disability, was used to assess motor impairment and functional performance. The ICF can facilitate a more comprehensive understanding of the effectiveness of bilateral movement training during stroke rehabilitation and an optimal bilateral movement training scheme for improving upper limb function.

According to the ICF framework, the outcome measure for the meta-analysis was divided into two domains: (1) motor impairment and (2) functional performance. The effect size of each outcome was computed by calculating the mean difference (MD), standard mean difference (SMD) and 95% confidence interval (CI), as appropriate. If a study did not provide the standard deviation (SD) of the MD or SMD, this value was estimated using the following formula, with the correlation coefficient (Corr) set to 0.8 [50]:

$$\mathrm{SD}=\sqrt{{SDpre}^{2}+{SD\;post}^{2}-2\times Corr\times SDpre\times SDpost}$$

The results of the meta-analysis were then visualized using a forest plot (Review Manager 5.3; The Nordic Cochrane Centre, Cochrane Collaboration, Copenhagen, Denmark). To compare the effects of BULT and UULT during different phases of stroke, the included studies were classified as acute (mean post-stroke duration<1 month), sub-acute (mean post-stroke duration>1month to <1 year), chronic (mean post-stroke duration>1 year) or not reported.

To investigate the influence of treatment dosage on the effect size estimates, meta- regression analyses were performed using STATA 12.0 (Stata Corporation, College Station, TX, USA).

### Publication bias

Egger’s test is more frequently used than other tests to detect publication bias in a meta-analysis [51]. Accordingly, Egger’s test[52] was used to detect the probability of publication bias in this study.

### Heterogeneity test

The Higgins I^2^ index was used to evaluate the heterogeneity of the studies. The I^2^ boundary was set at 50%. A random effect model was used when I^2^ >50%, indicating heterogeneity. A fixed effect model was used when I^2^ <50%, indicating homogeneity (51).

## Results

### Study identification

**Fig 1:**
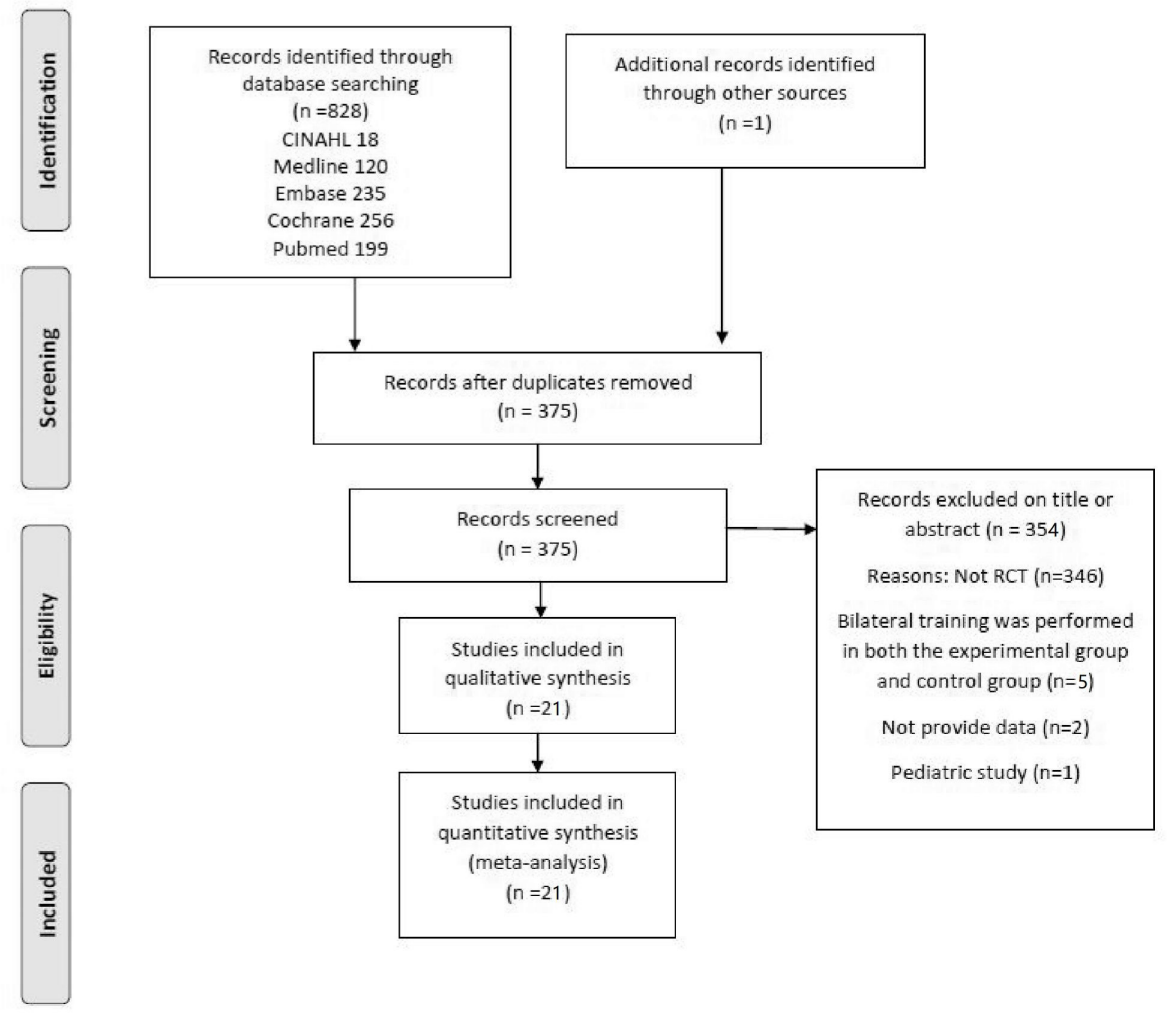
The preferred reporting items for systematic reviews and meta-analysis flowchart of study identification.

The search strategy yielded 828 citations on April 10, 2018. After excluding duplicated articles, 375 potentially relevant articles were subjected to further screening via a review of the abstracts. During this meta-analysis, the third reviewer made judgments on two of the screened articles [15, 20] that were ultimately included. Finally, 21 full-text articles with 842 subjects fulfilled the eligibility criteria of the review. Details of the studies’ identification, screening, eligibility and inclusion criteria are shown in Fig 1.

### Methodological quality

**Fig 2:**
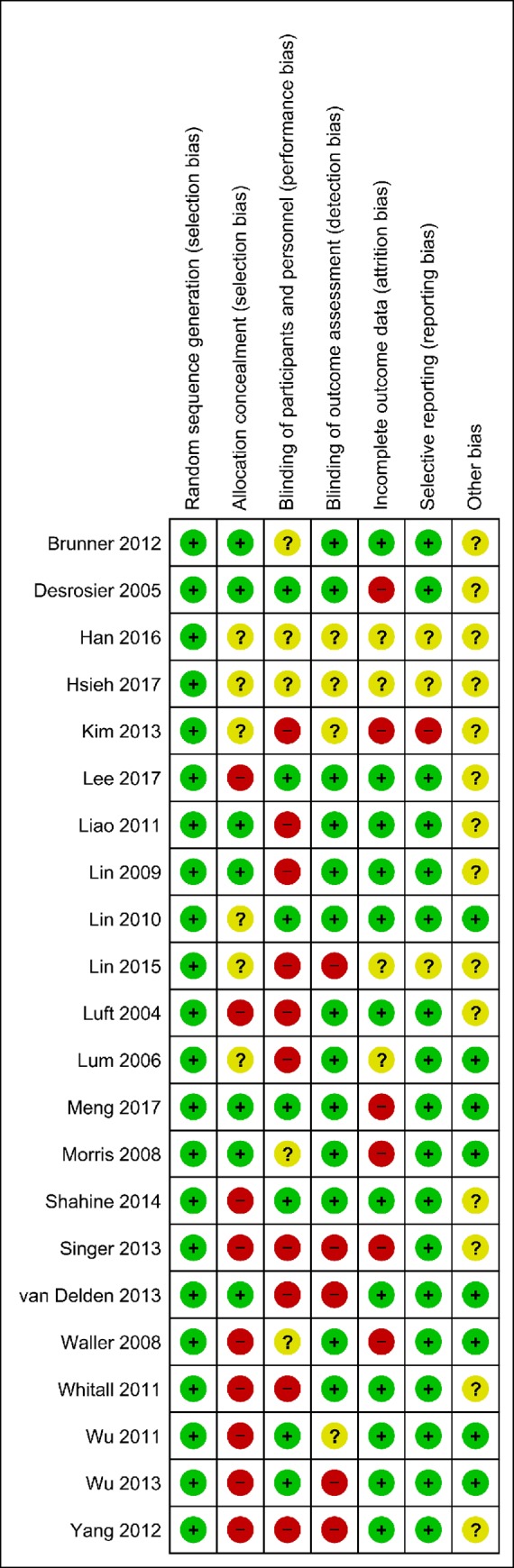
Risk of bias summary: A review of the authors' judgments about the risk of bias of each item in each included study.

Fig 2 presents the methodological quality of the included studies as evaluated using the Cochrane Collaboration risk of bias assessment tool [50].

### Characteristics of the subjects

Four studies [17, 21, 53, 54] compared the effects of BULT and UULT in subjects with acute stroke. Two studies [26, 46] compared the effects of BULT and UULT in people with sub-acute stroke. Thirteen studies [15, 16, 18, 19, 22–25, 28, 55–58] compared the effects of these rehabilitation strategies in subjects with chronic stroke. Two studies [20, 27] did not report details of the post-stroke duration. The demographic characteristics of the subjects and the characteristics of the studies are shown in Tables 2 and 3, respectively.

**Table 2:** Cohort characteristics of studies included in the meta-analysis (alphabetically list).

Author & Year	Gender	Age	Mean stroke duration(year)	Lesion type	Side affected	Baseline of FMA-UE
Brunner 2012 [53]	BULT:8/8; UULT:3/11	BULT:64.8±12.8; UULT:61.0±10.0	BULT:0.1±0.1; UULT:0.1 ±0.1	BULT:4/12; UULT:1/13	BULT:4/12; UULT:1/13	Not Reported
Desrosier 2005 [54]	BULT:11/9; UULT:11/10	BULT:72.2±10.8;UULT:74.3±10.1	BULT:0.09±0.09;UULT:0.10±0.09	BULT:1/19; UULT:0/21	BULT:13/7; UULT:10/11	BULT: 42.90±20.00; UULT: 47.00±16.10
Han 2016 [15]	BULT:8/5; UULT:9/3	BULT:78.8; UULT:72.9	BULT:6.92; UULT:6.48	Not Report	BULT:13/0; UULT:12/0	Not Reported
Hsieh 2017 [46]	BULT:5/11; UULT:8/7	BULT:49.28±10.90; UULT:52.87±10.40	BULT:0.21±0.14; UULT:0.18 ±0.09	BULT:8/8; UULT:8/7	BULT:8/8; UULT:4/11	BULT: 26.81±12.13; UULT: 29.07±16.12
Kim 2013 [20]	Not Reported	Not Reported	Not Reported	Not Reported	BULT:1/4; UULT:2/3	BULT: 24.4±5.2; UULT: 23.8±7.7
Lee 2017 [27]	BULT:6/9; UULT:5/10	BULT:57.33± 9.88; UULT: 54.60 ± 16.03	Not Reported	BULT:7/8; UULT:9/6	BULT:5/10; UULT:6/9	BULT: 48.73±16.42; UULT: 46.60±12.03
Liao 2012 [22]	BULT:4/6; UULT:3/7	BULT:55.51±11.17; UULT:54.56±8.20	BULT:1.99±1.12; UULT:1.09±0.68	Not Reported	BULT:4/6; UULT:3/7	BULT: 44.90±9.02; UULT: 39.60±11.27
Lin 2010 [18]	BULT:6/10; UULT:8/9	BULT:52.08± 9.60; UULT:55.50±13.17	BULT:1.16±1.06; UULT:1.09±1.1	Not Reported	BULT:9/7, UULT:8/9	BULT: 45.50±10.35; UULT: 49.75±12.10
Lin 2015 [19]	BULT:4/12; UULT:0/17	BULT:52.63±10.49; UULT: 57.47±10.29	BULT:2.31±1.59; UULT:1.82±1.81	BULT:9/7; UULT:6/11	BULT:4/12; UULT:0/17	BULT: 35.69±15.56; UULT: 38.71±19.98
Luft 2004 [23]	BULT:2/7; UULT:7/5	BULT:63.3±15.3; UULT:59.6±10.5	BULT:6.25; UULT:3.79	BULT:9/0; UULT:12/0	BULT:3/6; UULT:4/8	BULT: 29.60±12.25; UULT:28.30 ±4.41
Lum 2006 [26]	BULT:3/2; UULT:2/4	BULT:72.2±11.7; UULT:59.9±5.5	BULT:0.12±0.02; UULT:0.20±0.05	Not Reported	BULT:2/3; UULT:2/4	BULT: 39.20±4.30; UULT: 26.00±3.30
Meng 2017 [21]	BULT:30/34; UULT:33/31	BULT:55.38±6.97; UULT:55.19±7.82	BULT:8.87±2.69h; UULT:9.08±2.35h	BULT:14/50; UULT:19/45	BULT:29/35; UULT:31/33	BULT: 33.25±5.89; UULT: 32.86±5.11
Morris 2008 [17]	BULT:22/34; UULT:23/27	BULT:67.9±13.1; UULT:67.8±9.9	BULT:0.06±0.02; UULT:0.06±0.02	BULT:53/3; UULT: 44/6	BULT:27/29; UULT:27/23	Not Reported
Shahine 2014 [58]	BULT:19/21; UULT:17/19	BULT:61.4±5.5; UULT:62.7±3.1	BULT:2.6±1.8; UULT:3.0±1.6	Not Reported	BULT:23/17; UULT:21/15	BULT: 40.50±6.20; UULT: 38.50±6.10
Singer 2013 [57]	BULT:4/6; UULT:2/8	BULT:68.6±9; UULT: 68±16.4	BULT:4.33±4.02; UULT:5.33±4.12	Not Reported	BULT:5/6; UULT:5/5	BULT: 38.00±9.60; UULT: 30.50±12.80
van Delden 2013 [28]	BULT:8/11; UULT:3/16	BULT:62.6±9.8; UULT:56.9±12.7	BULT:7.8±4.9; UULT:11.1±6.8	Not Reported	BULT:11/8; UULT:11/8	BULT: 42.70±12.40; UULT: 39.00±10.30
Waller 2008 [56]	BULT:4/5; UULT:7/2	BULT:58.0±12.4; UULT:54.1±8.6	BULT:6.1±5.8; UULT:2.6±1.8	Not Reported	BULT:5/4; UULT:5/4	BULT: 35.22±12.30; UULT: 34.00±13.20
Whitall 2011 [25]	BULT:16/26; UULT:26/24	BULT:59.8±9.9; UULT:57.7±12.5	BULT:4.5±4.1; UULT:4.1±5.2	Not Reported	BULT:18/23; UULT:25/25	BULT: 32.30±2.20; UULT: 31.00±2.10
Wu 2011 [16]	BULT:4/18; UULT:6/16	BULT:52.22±10.72; UULT:55.19±2.50	BULT:1.33±1.15; UULT:1.48±1.04	Not Reported	BULT:10/12; UULT:12/10	Not Reported
Wu 2013 [24]	BULT:5/13; UULT:5/12	BULT:52.21±12.2; UULT:54.22±9.78	BULT:1.94±1.28; UULT:1.95±1.27	Not Reported	BULT:9/9; UULT:9/8	Not Reported
Yang 2012 [55]	BULT:3/4; UULT:2/5	BULT:51.4±10.9; UULT:50.8±6.1	BULT:1.23±0.48; UULT: 1.03±0.37	Not Reported	BULT:4/3; UULT:4/3	BULT: 41.90±3.90; UULT: 40.90±6.40

BULT, Bilateral Upper Limb Training; UULT, Unilateral Upper Limb Training; FMA-UE, Fugl-Meyer Assessment of Upper Extremity

**Table 3:** Characteristics of the included studies, including types, duration, frequency of intervention and outcome measures.

Author & Year	Intervention type for BULT and UULT	Duration of therapy	Outcome measures
Brunner 2012 [53]	BULT: Grip objects of different sizes and shapes, Fold a towel, Lift a glass, Press the keys of a keyboard, Point to a target, Catch a ball, Carry an object UULT: Same as bilateral group but performed with affected hand	144–160 mins per session; 4 sessions, 1/wk; 4 wks	ARAT, 9 Hole Peg Test, MAL
Desrosier 2005 [54]	BULT: Fold hand towels, Wipe the table, Sort buttons quickly, Roll out dough, Open and close various types of locks, Spoon out dry ingredients UULT: Passive and assisted movements of the affected arm, Putting blocks or cones in a pile, Unscrewing a light bulb, Shuffling playing cards, Putting a pillow in a pillow case, Tearing up sheets of paper	45 mins per session;15–20 session, 4/wk; 5 wks	FMA, Grip strength, BBT, Purdue Pegboard Test, Finger-to-Nose Test, TEMPA, MIF, FIM
Han 2016 [15]	BULT: Hanging a ring bilaterally, Cleaning a desk with a towel bilaterally, Drinking water bilaterally UULT: Same as bilateral group but performed with affected hand	Duration per session not given; 30 sessions, 5/wk 6wks	BBT; Shoulder and Elbow amplitude; Shoulder and Elbow Variability
Hsieh 2017 [46]	BULT: Approximately 1200 to 1600 repetition of passive and active bilateral forearm pronation/supination and wrist flexion/extension movement training, Filling a bottle from a fountain, transferring to the therapy room, and drinking water from the bottle, Wipe the table with a cloth, Folding towels and putting them in the drawers UULT: Same as bilateral group but performed with affected hand	90 min per session; 20 sessions, 5 /wk 4 wks	FMA; Grip Strength; BBT; SIS; FIM; mRS; Actigraphy
Kim 2013 [20]	BULT: Flower game, Paint game, Joint movement game, Reach game, Pong game, Circular pong, Pinball game, Hand ball games UULT: Same as the BRT, but only perform with the affected hand	90 min per session; 12 sessions, 2/wk 10wks	FMA; ROM of shoulder, elbow and wrist; Paint area; Travel distance; Area around straight line; Efficiency index
Lee 2017 [27]	BULT: 30 min of bilateral arm training (dishwashing, making coffee, typing, cutting fruit, and folding laundry), 30 min of general occupational therapy (neurodevelopmental treatment, stretching exercises, resistance movement) UULT: 60 min of general occupational therapy	60 min per session; 40 sessions, 5/wk 8wks	FMA; BBT; MBI
Liao 2012 [22]	BULT: 300 to 400 forearm cycles, totaling 600–800 repetitions of passive-passive mode and passive-active mode, 150–200 repetitions of resistance mode, 15 minutes of twisting a towel, turning a key in the lock, opening a jar, carrying heavy objects, using chopsticks, writing, folding clothes, picking up coins, turning a door knob UULT: Affected arm exercise or gross motor training, Muscle strengthening of the affected arm, Fine motor or dexterity training, Picking up telephone handset for listening, pulling out a drawer, Turning pages of a book, Writing, Using forks or safety knives for cooking, Opening a jar	90–105 mins per session; 20 session, 5/wk 4 wks	FMA; arm activity ratio; FIM; MAL; ABILHAND questionnaire
Lin 2010 [18]	BULT: lift 2 cups, stack 2 checkers, pick up 2 small dried beans, fold 2 towels, turn 2 large screws, manipulate 2 coins, use both hands to hold a sprinkler can to water plants UULT: neurodevelopmental techniques, trunk–arm control, weight bearing by the affected arm, fine motor tasks practice, practice on compensatory strategies for daily activities	120 mins per session; 15 sessions, 5/wk 3wks	FMA; FIM; MAL
Lin 2015 [19]	BULT: Bilateral isometric handgrip force training, gradually increased or decreased their grip strengths with both hands to track the trajectory of the targeted force. UULT: Routine clinical rehabilitation: strengthening, stretching, practicing of functional tasks, and coordination and weight bearing training of the hemiparetic upper limb	30 mins per session; 12 sessions, 3/wk 4 wks	FMA; WMFT; MAS; BI; FIM
Luft 2004 [23]	BULT: Eight times of pushed and pulled 2 T-bar handles sliding in the transverse plane bilaterally with auditory cues, in synchrony or alternation UULT: Thoracic spine mobilization, Scapular Mobilization, Weight bearing with the paretic arm, Opening a closed fist	60 mins per session; 18 sessions, 3/wk 6wks	FMA; WMFT; Shoulder Strength; Elbow Strength; UMAQS; fMRI
Lum 2006 [26]	BULT: 12 reaching movement (bilateral mode), Rhythmic circular movement (bilateral mode), Tone normalization and limb positioning UULT: NDT, Tone normalization and limb positioning	60 mins per session; 15 sessions, 4 wks	Ashworth scale; FMA; FIM; MSS; Motor Power examination
Meng 2017 [21]	BULT: Haptic perception Training, Bimanual coordination Training, Functional training of the hands UULT: Conventional rehabilitation training	120 mins per session; 20 sessions, 10 sessions/wk 2 wks	FMA; ARAT; AMP; RMT; CMCT
Morris 2008 [17]	BULT: Reaching, Forearm pronation and supination, Wrist extension, Grasp UULT: Same as bilateral group but performed with affected hand	20 mins per session; 30 sessions 5 sessions/wk 6 wks	ARAT; RMA; 9 Hole Peg Test; MBI; Hospital Anxiety and Depression Scale; Nottingham Health Profile
Shahine 2014 [58]	BULT: 5 min of pushing and pulling the handle symmetrically for total 3 times (in-phrase), 5 min of pushing one handle away from the body by one hand and pulling the other handle toward the body by the other hand in time with an auditory cueing for total 3 times (antiphase), 10 min of rest for total three times UULT: Assisted range of motion exercises, Strengthening exercises, Fine motor tasks practice	60 mins per session; 24 sessions, 3/wk 8wks	FMA; MEP
Singer 2013 [57]	BULT: Grasp and release of a cup, Pouring water into a cup, Sorting cards, Opening an envelope, Unscrewing a jar/bottle lid UULT: Same as the BAT, but only perform with the paretic hand	30 mins per session; 42 sessions, 7/wk 6 wks	FMA; Arm Motor Ability Test; Inter-hemispheric Inhibition
van Delden 2013 [28]	BULT: 3-minute movement periods interspersed with 5-minute rest periods total 21 minutes of active movements: move both hands simultaneously towards flexion/extension followed by a movement towards extension/ flexion following an auditory cue. UULT: Exercise therapy presented by the Royal Dutch Society of Physical Therapy and the Dutch Society of Occupational Therapy	60 mins per session; 18 sessions, 3/wk 6 wks	FMA; ARAT; Motricity Index; 9 Hole Peg Test; Erasmus mod. Nottingham; MAL; SIS
Waller 2008 [56]	BULT: The arms moving simultaneously (in phase)/alternately (antiphase) with auditory cuing at a preferred speed UULT: Thoracic spine mobilization with weight shifting, Scapular mobilization, Weight bearing with the affected arm, Opening the hand with finger extension	60 mins per session; 18sessions, 3/wk 6wks	FMA; WMFT
Whitall 2011 [25]	BULT: The arms moving simultaneously (in phase)/alternately (antiphase) with auditory cueing at a preferred speed UULT: Thoracic spine mobilization with weight shifting, Scapular mobilization, Weight bearing with the affected arm, Opening the hand with finger extension	60 mins per session; 18 sessions, 3/wk 6wks	FMA; WMFT; SIS; Isokinetic Strength; Isometric Strength; fMRI
Wu 2011 [16]	BULT: Lifting 2 cups, Picking up 2 pegs, Grasping and releasing 2 towels, Wiping the table with 2 hands UULT: 75% functional task practice for hand function, UE coordination, balance, stretching, and weight bearing of the affected UE, 25% compensatory practice on functional tasks with the unaffected UE or both UEs	120 mins per session; 15 sessions, 5/wk 3wks	Kinematic variable; WMFT; MAL
Wu 2013 [24]	BULT: The paretic arm moved the handle independently, The paretic arm moved the handle against a resistance determined by the therapist through the entire movement UULT: Weight bearing, Stretching, Strengthening of the affected arm, Coordination tasks, Unilateral and bilateral fine motor tasks, Balance activities	90–105 mins per session;20 sessions, 5/wk 4wks	Kinematic variable; WMFT; MAL; ABILHAND Questionnaire
Yang 2012 [55]	BULT: 75–80 min of robot-assisted Training, 15–20 min of functional task practice included reaching to move a cup, grasping and releasing blocks, picking up coins wiping a table with two hands, picking up two pegs, opening a jar with one hand stabilizing while the other hand manipulated, 5 min of tone normalization for the arm UULT: Same as the BRT, but only perform with the affected hand	90–105 mins per session; 20 sessions, 5/wk 4wks	FMA; MRC; grip strength; MAS

AMP, motor-evoked potentials amplitude; ARAT, Action Research Arm Test; BATRAC, Bilateral Arm Training with Rhythmic Auditory Cueing; BAT, Bilateral Arm training; BBT, Box and Block Test; BULT: Bilateral Upper Limb Training; CMCT, central motor conduction time; FIM, Functional Independence Measure; FMA-UE, Fugl-Meyer Assessment of Upper Extremity; fMRI, functional magnetic resonance imaging; MAS: Modify Ashworth Scale; MBI, Modify Barthel Index; MIF, Mesure de l'independance fonctionnelle; MRC, Medical Research Council; mRS: modified Rankin Scale; MSS, Motor Statue Score; RAP, Rehabilitation Activities Profile; RMA, Rivermead Motor Assessment upper-limb scale; RMT, rest motion threshold; ROM, Range of Motion; SIS, Stroke Impact Scale; TEMPA: Test d'Evaluation des Membres Supérieurs de Personnes Agées; TOT, Task-oriented training; UMAQS, University of Maryland Arm Questionnaire; UULT: Unilateral Upper Limb Training; WMFT, Wolf Motor Function Test

### Characteristics of the intervention

Nine studies [15–18, 21, 27, 53, 54, 57] compared the effects of bilateral functional task training (e.g., folding a towel, lifting two cups and picking up two pegs bilaterally) and unilateral task-related training. Four studies [17, 21, 53, 54] compared these effects in people with acute stroke. Two studies [16, 18]compared the effects of bilateral functional task training and dose-matched neurodevelopmental therapy, weight-bearing exercises and unilateral functional task training. Two studies [15, 57] compared the effects of bilateral functional task training and unilateral functional task training in people with chronic stroke. Lee and colleagues [27] investigated the combined effects of 30 minutes of bilateral functional arm training and 30 minutes of standardized occupational therapy, which included neurodevelopmental therapy, stretching exercises, resistance training and fine movement training of the affected upper limb. The outcomes of combined therapy were then compared with those observed after 60 minutes of standardized occupational therapy.

Seven studies [19, 20, 22, 24, 26, 46, 55] explored the effects of bilateral robotic-assisted or resistance training on upper limb motor function after stroke. Three studies [20, 24, 55] compared the effects of 90 minutes of bilateral robotic-assisted training and of 90 minutes of unilateral robotic-assisted training. Three studies [19, 22, 26] compared the effects of bilateral robotic-assisted training and of dose-matched unilateral functional task training in subjects with chronic stroke. Hsieh et al. [46] compared a combination of robotic-assisted priming and task-oriented training with task-oriented training alone on the affected upper limbs of patients with sub-acute stroke.

Five studies [23, 25, 28, 56, 58] compared the effects of bilateral arm training involving rhythmic auditory cueing with the effects of dose-matched unilateral upper limb training, which included neurodevelopmental therapy, upper limb mobilization, strengthening exercises and fine movement training.

### Meta-analysis

Based on the measurement items categorized by the previous studies [59, 60], this review compared the overall effects of BULT and UULT on (1) improved motor impairment by pooling the results of the FMA-UE and on (2) functional performance by pooling the results of the WMFT, ARAT and the box and block test (BBT). The details of these outcome measures are presented in Table 4.

**Table 4:** Pooled assessments used to conduct the meta-analysis.

Assessment tool		Description
Motor Impairment	FMA-UE	FMA-UE is a stroke-specific assessment tool to measure the upper limb motor impairment [61], which included shoulder-arm, wrist, hand and coordination. There were 33 items, which scoring on an ordinal scale from 0 to 2. The total score was ranged from 0 to 66.
Functional Performance	WMFT	WMFT is used to assess the upper limb motor function in people with stroke [62], which included 2 strength-based tasks and 15 functional-based tasks. The 15 function-based tasks were assessed by the time taken to complete each task and the quality rating of the use of the affected hand in attempting each task. The functional task was graded from 0 to 5. The total score was ranged from 0 to 75.
	ARAT	ARAT is used to assess the upper limb function of grasping, gripping, pinching and gross arm movement [63]. This ordinal scale consists of 19 items. The quality of the performance on each item was rated from 0 to 3 points. The total score was ranged from 0 to 57.
	BBT	BBT is a measure of gross manual dexterity for handicapped people [64]. The test will count the number of wooden blocks that can be transported form one compartment of a box to another within 1 min. The more block being transported indicated a better functional performance.

ARAT, Action Research Arm Test; FMA-UE, Fugl-Meyer Assessment of Upper Extremity; BBT, Box and Block Test; WMFT, Wolf Motor Function Test

The meta regression indicated that the number of training sessions (p = 0.947), total duration of training (p = 0.217) and duration of training per session (p = 0.316) had no significant impact on the effect size of FMA-UE.

### Publication bias

**Fig 3:**
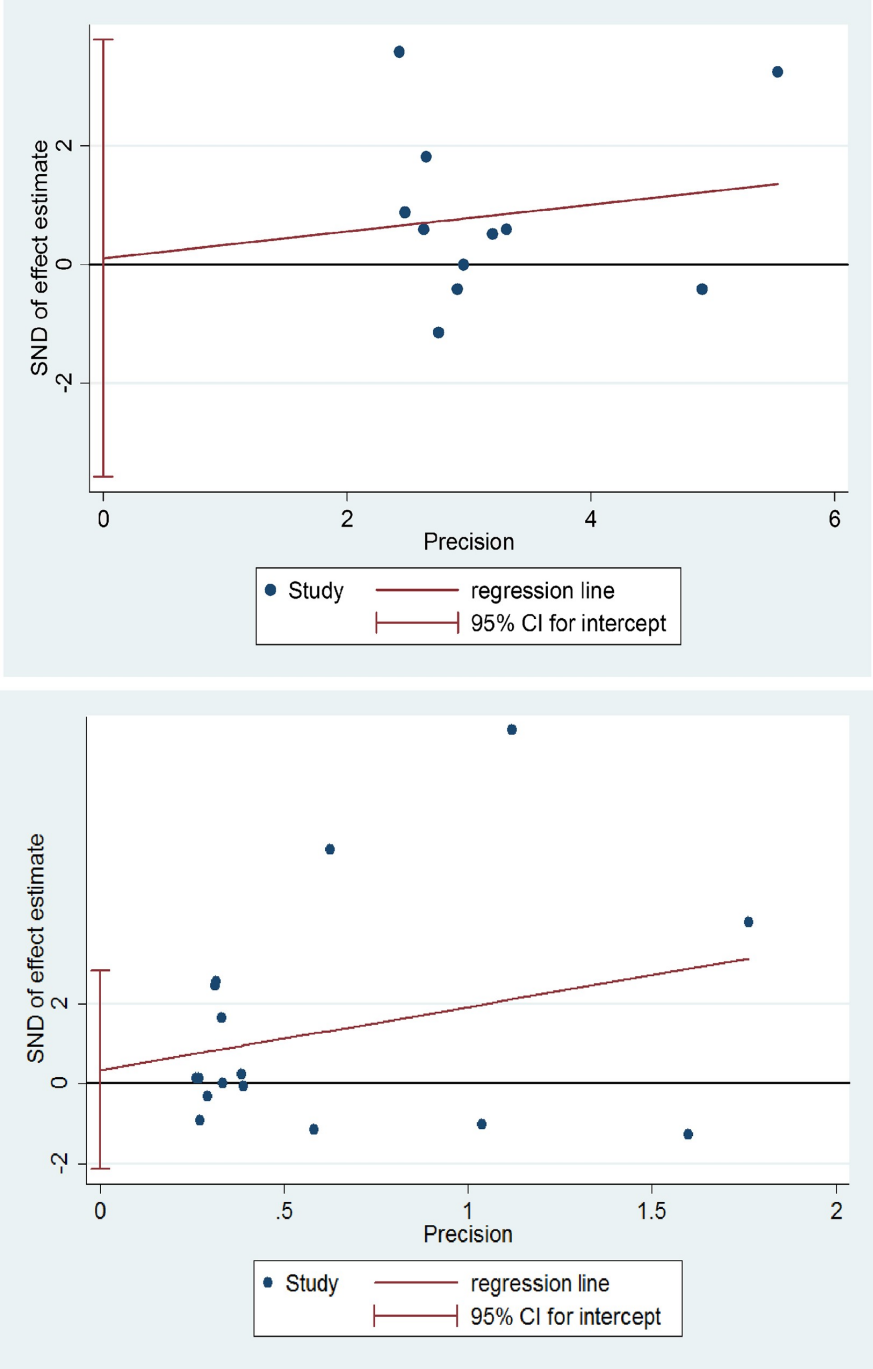
Results of Egger’s test for the publication bias in motor impairment (above) and functional performance (below). SND: standard normal deviation, CI: confidence interval.

According to the results of Egger’s test (Fig 3), no significant publication bias was observed in the meta-analysis of FMA-UE (p = 0.774) or of WMFT, ARAT or BBT (p = 0.950).

### Outcomes on motor impairment

**Fig 4:**
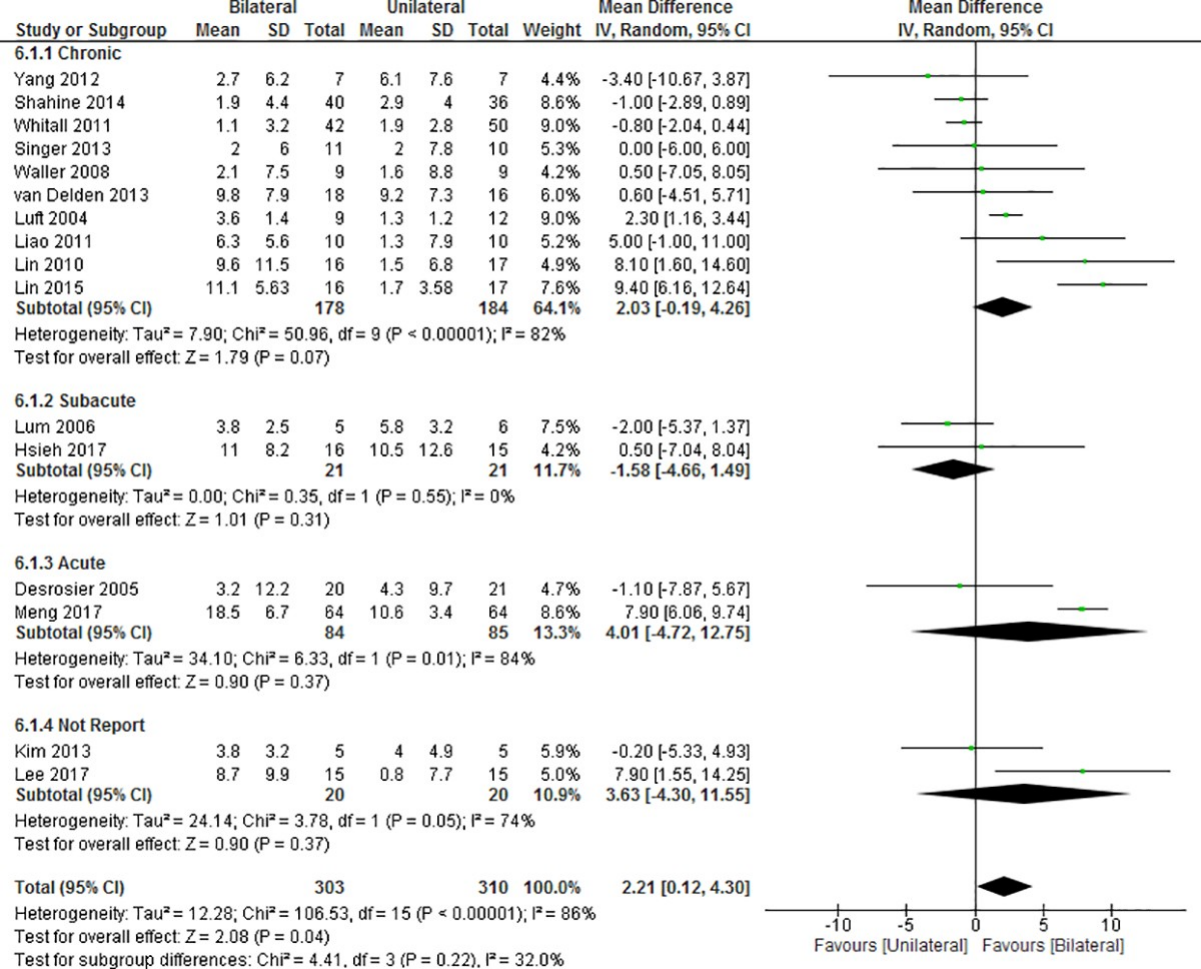
Differences in the mean (95%CI) effect of BULT relative to UULT in terms of FMA-UE, using pooled by pooling data from 16 studies. CI: confidence interval, IV: inverse variable, SD: standard deviation.

In 16 studies [18–23, 25–28, 46, 54–58], the FMA-UE was used to measure improvements in stroke-induced motor impairment (Fig 4). The meta-analysis revealed a significantly greater improvement in motor impairment in the BULT group, compared with the UULT group (MD = 2.21, 95%CI: 0.12 to 4.30, p = 0.04; I^2^ = 86%, p<0.001). However, no significant improvements were observed with BULT when compared with UULT in the subgroups according to post-stroke duration (chronic: MD = 2.03, 95%CI: -0.19 to 4.26, p = 0.07; I^2^ = 82%, p<0.001; subacute: MD = -1.58, 95%CI: -4.66 to 1.49, p = 0.31; I^2^ = 0%, p = 0.55; acute: MD = 4.01, 95%CI, -4.72 to 12.75, p = 0.37; I^2^ = 84%, p = 0.01; not report: MD = 3.63, 95%CI: -4.30 to 11.55, p = 0.37; I^2^ = 74%, p = 0.05).

### Functional performance outcomes

In this study, improvements in functional performance were assessed using the time component of WMFT and the WMFT, ARAT and BBT scores.

**Fig 5:**
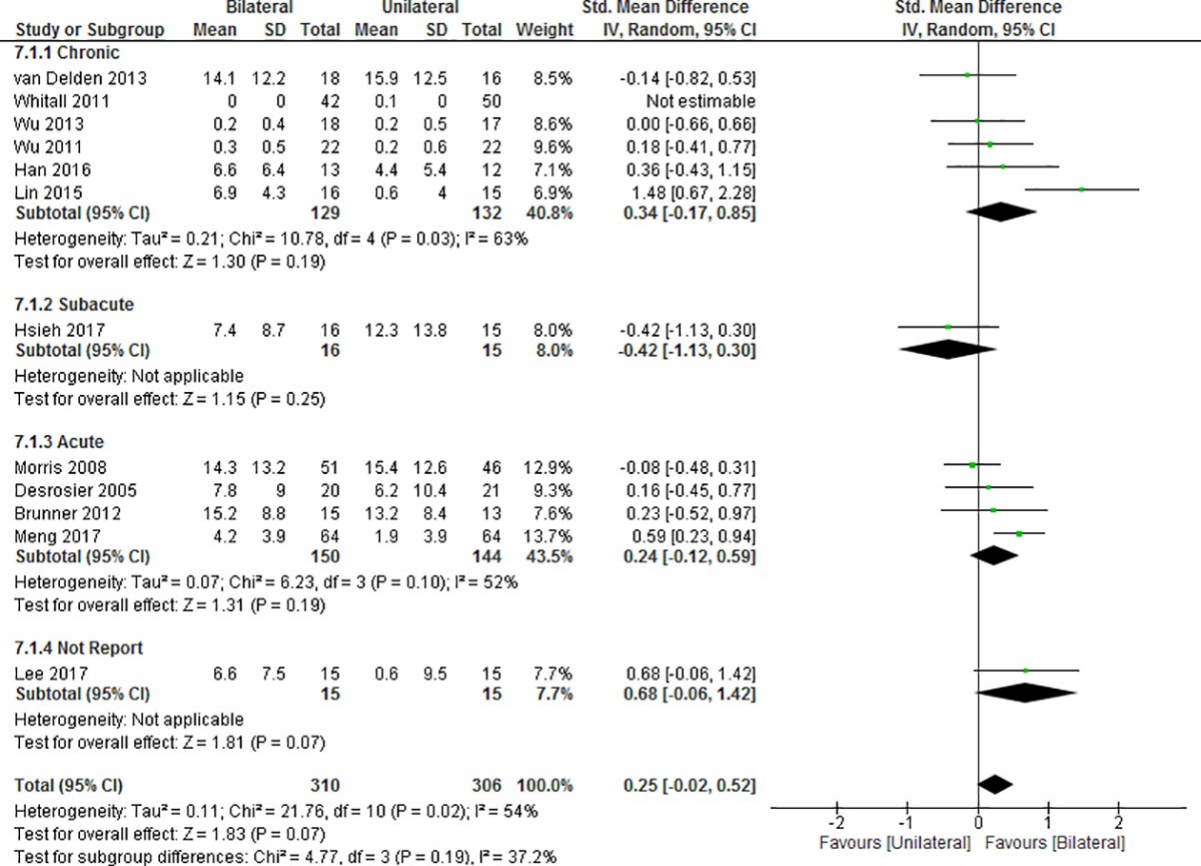
Difference in the mean (95%CI) effect of BULT related to UULT on the measures of WMFT, ARAT and BBT in data pooled from 12 studies. CI: confidence interval, IV: inverse variable, SD: standard deviation.

The improvement in functional performance was measured by the functional ability scores of the WMFT, ARAT and BBT in 12 studies (15–17, 19, 21, 24, 25, 27, 28, 46, 53, 54) (Fig 5). Among them, four studies [17, 21, 28, 53] investigated the improvement in functional ability by ARAT, four studies [16, 19, 24, 25] measured the functional ability score of the WMFT and four studies (15, 27, 46, 54) measured BBT. No significant difference was observed between BULT and UULT in terms of improvements in the score component of the WMFT (SMD = 0.25, 95%CI: -0.02 to 0.52; p = 0.07; I^2^ = 54%, p = 0.02). Similarly, BULT did not yield significant improvement when compared with UULT in any of the post-stroke duration subgroups (chronic: SMD = 0.34, 95%CI: -0.17 to 0.85, p = 0.19; I^2^ = 63%, p = 0.03; subacute: SMD = -0.42, 95%CI: -1.13 to 0.30, p = 0.25; acute: SMD = 0.24, 95%CI: -0.12 to 0.59, p = 0.19; I^2^ = 52%, p = 0.10; not report: SMD = 0.68, 95%CI: -0.06 to 1.42, p = 0.07).

**Fig 6:**
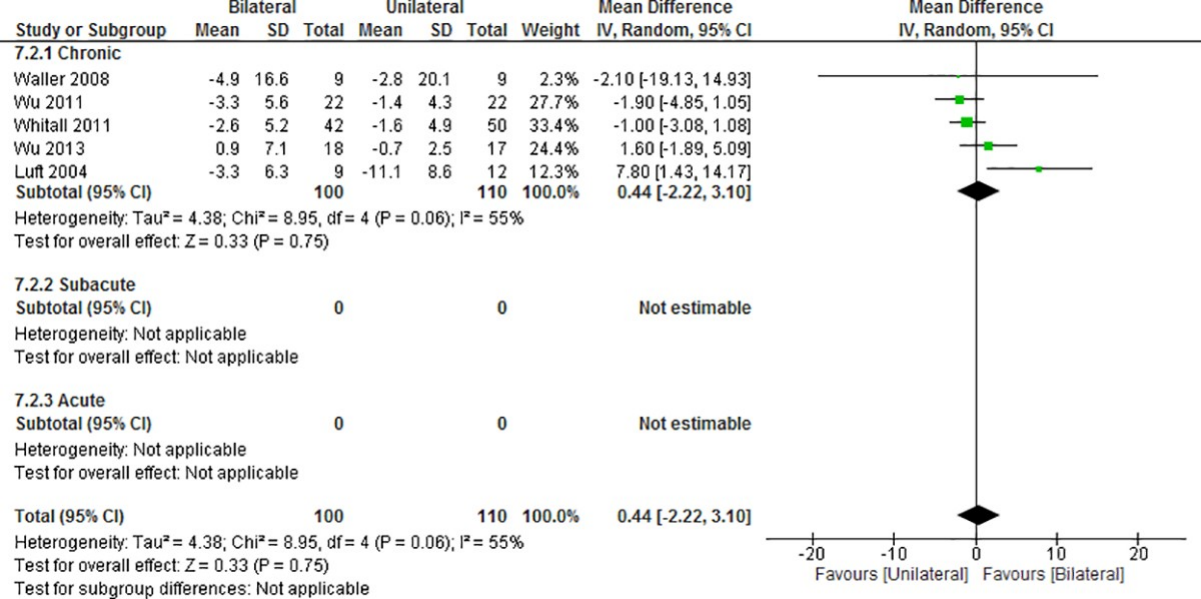
Difference in the mean (95%CI) effect of BULT relative to UULT on the time component of WMFT in data pooled from 5 studies. CI: confidence interval, IV: inverse variable, SD: standard deviation.

Five studies [16, 23–25, 56] were used to evaluate the effect on improvement in the time required for the WMFT (Fig 6). A comparison of BULT and UULT revealed no significant difference in the time component of the WMFT (MD = 0.44, 95%CI: -2.22 to 3.10; p = 0.75; I^2^ = 55%, p = 0.06). Similarly, BULT did not yield a no significant improvement over UULT in the subgroups (chronic: MD = 0.44, 95%CI, -2.22 to 3.10, p = 0.75; I^2^ = 55%, p = 0.06).

## Discussion

This is the first systematic review and meta-analysis to compare the effects of BULT and UULT on motor impairment and functional performance in people with stroke after excluding CIMT. The exclusion of CIMT may provide a more realistic overview of the effects of actual rehabilitation efforts. The meta-analysis examined the pooled results of 21 RCTs including 842 subjects with stroke. According to this analysis, BULT yielded significantly greater improvement in the FMA-UE, compared to UULT. However, no significant differences in the change of functional performance as indicated by the WMFT, ARAT and BBT were observed between BULT and UULT.

### Motor impairment

Our results are partially consistent with the findings of a review by Coupar et al. [30]. In that review [30] of two sets of four studies, the authors found that BULT was more effective than the usual care in terms of improving the FMA-UE scores in people with stroke. However, Coupar and colleagues [30] found no differential effects between BULT and UULT in terms of improving the FMA-UE score [17, 26, 65, 66]. Our inclusion and exclusion criteria enabled us to include 4 studies [17, 23, 26, 54]from the review by Coupar and 12 other RCTs in our meta-analysis. Thus, our review may have elicited more robust estimations of the overall effects of BULT and UULT on improvments in the FMA-UE.

Two reviews [33, 40] found that BULT and UULT yielded similar reductions in motor impairment in people with stroke, as indicated by the FMA-UE score. However, these results should be interpreted cautiously because van Delden [25, 42, 65, 66] and Lee [25, 42, 65, 67] each reviewed four studies to estimate the effects of BULT and UULT on the FMA-UE. In contrast, our meta-analysis on FMA-UE was based on 16 RCTs, and included a larger sample size. Our review would therefore have a stronger power to detect a difference between BULT and UULT in terms of an improved FMA-UE score. The reviews by Lee [40] and van Delden [33] also included two studies [42, 66] that compared the effects of BULT and CIMT on improved FMA-UE scores. In our study, we only compared BULT with task-related training. This heterogeneity in the study samples explains the different conclusions of our review and the other two reviews.

Our review demonstrated a significantly greater improvement in the FMA-UE scores of people with stroke after BULT, compared with UULT. Moreover, we found no significant association of the training dosage with the improvements in FMA-UE. Therefore, the different levels of improvement between BULT and UULT was mainly attributable to the types of intervention (BULT/UULT). The excitability typically decreases in the lesional cerebral hemisphere and increases in the contralesional hemisphere [68–70]. TMS studies [67, 71–73] have indicated that this restoration of interhemispheric imbalance positively correlates with motor recovery after stroke. Accordingly, the different levels of improvement in the FMA-UE scores after BULT and UULT may be related to the use of different mechanisms to facilitate the reorganization in the lesional hemisphere. UULT is based on the principle of activation in the lesional hemisphere via assisted or resisted unilateral training of the paretic limb [8, 74, 75]. Compared with UULT, BULT activates similar neural networks in the bilateral hemispheres during the simultaneous activation of homologous muscle groups [14, 76–78]. Studies have shown that BULT can activate the distributed corticospinal pathway bilaterally via ipsilateral corticospinal fibers [79–81], contralateral corticospinal fibers [82–84] and the corpus callosum[85, 86]. Compared with UULT, BULT may evoke greater activation of the lesional hemisphere by recruiting more neural pathways [14]. In the BULT group, increased activation in the lesional hemisphere might have led to greater improvements in motor impairment, as indicated by the FMA-UE scores.

### Functional performance

Consistent with the findings of previous reviews [33, 40], our meta-analysis indicated that BULT was not superior to UULT in terms of improving the functional performance of people with stroke, as measured by the WMFT, ARAT and BBT. Although we observed a significantly greater improvement in the FMA-UE with BULT than with UULT, we observed no significant differences in functional performance between the training strategies. Buchner [87] reported a non-linear relationship between leg strength and gait speed in people with stroke. This curve revealed a positive slope that gradually decreased to zero; in other words, the curve eventually reached a plateau, as the leg strength increased. A muscle strength threshold is required to perform each type of activity. However, increased strength does not result in an improved gait speed until a certain threshold is reached [88]. Similarly, the improvement in the FMA-UE score and the functional performance may also exhibit a non-linear relationship. Although previous studies [62, 89–91] reported a moderate to good correlation between the FMA-UE and functional performance-related scales (e.g., WMFT, ARAT and BBT), the non-linear relationship observed between these fators in our meta-analysis may reflect an inability of the stroke survivors to achieve the motor control threshold needed to perform the functional tasks. Although the FMA-UE improved to a significantly greater level in the BULT group when compared with the UULT group, this significant improvement may have been insufficient to yield a significant improvement in functional performance.

Our meta-analysis revealed that bilateral functional task training tended to yield a larger SMD for improving functional performance, compared to bilateral robotic-assisted training and bilateral arm training with auditory cueing. However, this result should be interpreted cautiously because although our meta-analysis included 9 of studies [15–18, 21, 27, 53, 54, 57] on bilateral functional task training (the largest proportion), it also included only 7 studies [19, 20, 22, 24, 26, 46, 55] of bilateral robotic-assisted training and 5 studies [23, 25, 28, 56, 58] of bilateral arm training with auditory cueing. Therefore, the inclusion of more studies of bilateral robotic-assisted training and bilateral arm training with auditory cueing would support more robust conclusions regarding the effects of the different types of BULT. Moreover, we used WMFT, ARAT and BBT to estimate improvements in functional performance. The items included in these evaluation tools, such as gripping objects, folding a towel and lifting objects, are similar to the components of bilateral functional task training. Accordingly, there may be a stronger learning effect in the studies that investigated bilateral functional task training, compared to those that investigated bilateral robotic-assisted training and bilateral arm training with auditory cueing.

This systematic review had several limitations. First, we only examined 21 studies with 842 subjects. This sample size may not have been sufficiently large to detect significant differences in the functional performance outcomes. Second, the results of this review may not be generalizable to all stroke survivors. In addition to the 13 studies [15, 16, 18, 19, 22–25, 28, 55–58] of people with chronic stroke included in this study, only 4 studies [17, 21, 53, 54] of people with acute stroke and 2 studies [26, 46] of people with sub-acute stroke investigated the effects of BULT. Therefore, the true effect of BULT may be underestimated because of the small number of included non-chronic stroke studies. The inclusion of more studies with subjects in different phases of stroke would increase the generalizability of our conclusions. Third, most studies of Asian populations reported a significant improvement after BULT, compared with UULT. By contrast, most studies conducted in western countries reported insignificant differences between BULT and UULT. However, the reason underlying this discrepancy remains unclear. In future reviews, clear methodological information and a larger sample size may help to explain this phenomenon. Fourth, we only evaluated the immediate effects of the outcome measures. Our meta-analysis did not calculate the carry-over effects of BULT and UULT in terms of improving the FMA-UE, WMFT, ARAT and BBT score, as only 29% of the studies [17, 26, 28, 46, 53, 57] included in our review provided data from the follow-up assessments. Thus, our findings may not provide sufficient power to estimate the carry-over effects of BULT and UULT in people with stroke. These carry-over effects should be explored further. Fifth, the studies included in this review did not classify the severity of the motor impairment experienced by people with stroke. Therefore, we did not have sufficient information to analyze the effects of BULT and UULT with respect to the severity of the motor impairment.

## Conclusions

Both BULT and UULT can help to improve motor impairment and functional performance after stroke. Notably, BULT was superior to UULT in terms of improving motor impairment after stroke, as measured by the FMA-UE. However, BULT and UULT yielded similar effects on functional performance in people with stroke, as measured by the WMFT, ARAT and BBT.

## Supporting information

S1 File Original data of the review. (XLSX)
